# A 57 kB Genomic Deletion Causing CTNS Loss of Function Contributes to the *CTNS* Mutational Spectrum in the Middle East

**DOI:** 10.3389/fped.2019.00089

**Published:** 2019-03-21

**Authors:** Maryam Najafi, Dor Mohammad Kordi Tamandani, Anoush Azarfar, Zeineb Bakey, Farkhondeh Behjati, Dinu Antony, Isabel Schüle, Simin Sadeghi-Bojd, Ehsan Ghayoor Karimiani, Miriam Schmidts

**Affiliations:** ^1^Genome Research Division, Human Genetics Department, Radboud University Medical Center Nijmegen and Radboud Institute for Molecular Life Sciences, Nijmegen, Netherlands; ^2^Department of Biology, University of Sistan and Baluchestan, Zahedan, Iran; ^3^Department of Pediatrics, Faculty of Medicine, Mashhad University of Medical Sciences, Mashhad, Iran; ^4^Center for Pediatrics and Adolescent Medicine, Faculty of Medicine, University Hospital Freiburg, Freiburg University, Freiburg, Germany; ^5^Genetics Research Center, University of Social Welfare and Rehabilitation Sciences, Tehran, Iran; ^6^Children and Adolescents Health Research Center, Resistant Tuberculosis Institute, Zahedan University of Medical Sciences, Zahedan, Iran; ^7^Next Generation Genetic Polyclinic, Mashhad, Iran; ^8^Razavi Cancer Research, Razavi Hospital, Imam Reza International University, Mashhad, Iran

**Keywords:** Cystinosis, *CTNS* deletion, Middle East population, Iran, tubulopathy

## Abstract

**Background:** Nephropathic Cystinosis, the most common cause of renal Fanconi syndrome, is a lysosomal transport disorder with an autosomal recessive inheritance pattern. A large number of mutations in *CTNS* have been identified as causative to date. A 57 kb deletion encompassing parts of *CTNS* is most commonly identified in Caucasians but this allele has not been identified in individuals of Eastern Mediterranean, Middle Eastern, Persian, or Arab origin to date.

**Methods and Results:** Implementing whole exome sequencing (WES) in a consanguineous Iranian family, we identified this large deletion affecting *CTNS* in a patient initially presenting with hypokalemic metabolic alkalosis symptoms and considerable proteinuria.

**Conclusion:** We show WES is a cost and time efficient genetic diagnostics modality to identify the underlying molecular pathology in Cystinosis individuals and provide a summary of all previously reported CTNS alleles in the Middle east population. Our work also highlights the importance to consider the 57-kb deletion as underlying genetic cause in non-European populations, including the Middle East. Limited diagnostic modalities for Cystinosis in developing countries could account for the lack of previously reported cases in these populations carrying this allele. Further, our findings emphasize the utility of WES to define genetic causes in clinically poorly defined phenotypes and demonstrate the requirement of Copy number variation (CNV) analysis of WES data.

## Introduction

Cystinosis is an autosomal recessive lysosomal storage disorder (general incidence of 1:100,000–200,000 live births) caused by mutation of the *CTNS* gene (1, 2). *CTNS* locates to chromosome 17p13, contains 12 exons of which ten exons, exon 3–12, encode for Cystinosin, a protein facilitating cysteine-proton co-transport out of lysosomes (3, 4). Impaired Cystinosin function leads to the build-up of intralysosomal cystine crystals in cells of many different organs, notably the bone marrow, lymph nodes, kidney, cornea, thyroid, liver and spleen (5). Based on the age at onset and the severity of clinical disease, three cystinosis subtypes are distinguished: the most severe subtype is nephropathic cystinosis (OMIM#219800) which is also known as infantile onset renal tubular fanconi syndrome that affects proximal renal tubular function and results in failure to thrive, polyuria, and polydipsia with a disease onset between age 6 and 18 months on average. This is also the most frequent manifestation of cystinosis accounting for up to 95% of all cases (6). End-stage renal disease (ESRD) occurs often around 10 years of age if untreated (1). The two other milder subtypes, the nephropathic juvenile (late onset) subtype (OMIM# 219900) and the ocular non-nephropathic adult subtype (OMIM#219750) are rarely diagnosed before adult-hood (7, 8). Genotype-phenotype correlations suggest that milder adult-onset phenotypes may occur as a result of at least one milder mutation in comparison to mutations identified to cause the early-onset phenotype (9). Early diagnosis is important especially in nephropathic cystinosis as treatment with aminothiol cysteamine can slow down progression of the renal phenotype (10).

Commonly followed diagnostic approaches for cystinosis include measurement of leukocyte cystine, detection of corneal cystine crystals by the slit lamp examination and molecular testing of the relatively small *CTNS* gene (11). Nonetheless, in some developing countries, cystinosis diagnosis is still significantly delayed or may not happen due to limited access to healthcare and likewise limited diagnostic resources. In most instances, a cystinosis diagnosis in these countries is made due to (often repetitive) ophthalmological examinations showing accumulation of cystine crystals in the cornea. To date, ~120 causative *CTNS* mutations including small deletions or insertions, missense, nonsense, and splice site mutations in the coding regions or adjacent intronic regions as well as in the *CTNS* gene promoter have been described (www.hgmd.cf.ac.uk). Of note, the most common mutation is a large deletion, affecting the first 10 exons of the *CTNS* gene in either homozygous or heterozygous form. This variant is particularly frequent in Caucasians, accounting for up to 76% of all cases in this group of individuals (12). The breakpoints of this 57,257 base pair deletion locate in exon 10 of *CTNS* and intron 2/3 of *TRPV1*, resulting in deletion of large parts of *CTNS*, all of *CARKL/SHPK* and the two non-coding exons of *TRPV1* (13). However, in other populations, this deletion appears a lot less frequent and previous reports did not identify this allele in cystinosis cases from Middle Eastern countries including Egypt, Iran, Jordan, Turkey, and Saudi Arabia (14).

In the present study, we used Whole Exome Sequencing (WES) to identify the underlying molecular cause in an Iranian subject with metabolic alkalosis. Interestingly, we identified the previously described 57 kB deletion encompassing *CTNS* exon 1–10.

## Methods

### DNA Samples

Consent forms were obtained from all the participants of this study. Ethical approval was obtained from the Ethics committee of Mashhad University of medical (IR.MUMS.REC.1395.534) and samples were processed in Nijmegen under the Diagnostic Innovation Program. Written informed consent from the parents or legal guardians of the patients/participants for the publication of their data. Genomic DNA was extracted from EDTA blood using standard salting out method. The concentration of DNA was measured by Qubit 2.0 (life technologies, Carlsbad, CA, USA).

### Whole Exome Sequencing

Two micrograms of DNA from the index case was subjected to WES at Novogene, Hongkong. Illumina HiSeq 2500. Exome capture was performed using Agilent SureSelect Human All Exon V6 Kit; sequencing was undertaken on an Illumina HiSeq 2500 Genome Analyzer machine. Paired-end sequencing resulting in sequences of 150 bases from each end of the fragments with a fraction_of_target_covered_with_at_least_10x of 99.5%, of at least 20x of 98.1%, of at least 50x of 87.7% and of 100x of 44.3%. UCSC hg19 was used as a reference genome. VarScan version 2.2.5 and MuTec and GATK Somatic Indel Detector were used to detect SNV and InDels, respectively. Data was filtered for MAF < 1% in public control databases such as dbSNP, ExAc, and gnomAD (gnomAD, http://gnomad.broadinstitute.org). subsequently, variants occurring with MAF > 1% in the Iranome (contains 800 healthy individuals) (http://www.iranome.ir) were also excluded. The remaining variants were filtered for known disease causing genes with emphasis on diseases compatible with the patient phenotype (renal tubular disorders). We prioritized homozygous variants due to the autosomal recessive inheritance pattern of disease and known consanguinity in the family. This did not reveal any variant in a gene previously associated with a tubular disorder. We therefore proceeded to visual inspection of the BAM file for known tubuluopathy genes and detected a large homozygous deletion encompassing most of *CTNS*.

### PCR and Sanger Sequencing

The visually identified large homozygous deletion encompassing *CARKL/SHPK* and the first 10 exons of *CTNS* found in the BAM file of the index case was confirmed using PCR and subsequent Sanger sequencing. Primers were designed such as the forward primer located in *TRPV1* and the reverse primer located in intron 10 of CTNS gene. AmpliTaq mastermix (Applied Biosystems) was used according to the manufacturer's protocol. A standard amplification program was used (initial denaturation at 95°C for 10min; followed by 35 cycles of denaturing at 94°C for 30 s, annealing at 58°C for 30 s, and extension at 72°C for 3 min and final extension at 72°C for 7 min). PCR products were cleaned using ExoSAP-IT® (USB, Cleveland, Ohio, USA) and subsequently submitted for bidirectional Sanger sequencing using a 3730XL DNA analyzer (ABI, Foster City). Primers sequences are available on request.

## Results

Our index case was a 1-year-old boy who was the third child of consanguineous healthy parents of Persian ethnicity from Iran. He was born after term gestation with birth weight of 3,200 g (P25-50) and a length of 49.2 cm (P25-50); the pregnancy and delivery were uncomplicated. At the age of nearly 5 months, he was hospitalized due to recurrent vomiting, polydipsia, and polyuria, failure to thrive was noted since the 4th month of life with a length of 62.2 cm (<P3.) weighing 6,800 g (<P3.) at the age of 8 months. On admission, urine analysis (dip stick) revealed a specific weight of 1,004, urine pH of 5, glucosuria (+++), proteinuria (+), and ketonuria (+) with no bilirubin, nitrite, or urobilinogene present. Urine microscopy did not reveal significant hematuria or leucocytes in the urine. Chemical urine analysis, for example for protein quantification and protein subtype identification could not be performed in the admitting center. Venous blood pH was 7.48 (normal range 7.35–7.45), HCO_3_ 16.7 mmol/L (normal range 22–26 mmol/L), pCO_2_ 22.6 mmHg (normal range 35–45 mmhg), pO_2_ 55.3 mmHg (normal range >30 mmHg), and O2 saturation was found to be 91.1%. Serum sodium was within normal ranges (140 mmol/L) but hypokalemia (potassium 2.4 mmol/L, normal range 3.6–5.4 mmol/L) was noted. Creatinine, ferritine, vitamin D, and TSH were within the normal range. Abdominal ultrasound did not reveal any abnormalities in the liver, gallbladder, pancreas, spleen, kidneys, or bladder. The biochemical findings of mild hypokalemic alkalosis (possibly with a respiratory component indicated by low CO2 e.g., due to screaming child) together with severe growth retardation, polydipsia and polyuria as well as the urine findings suggested a renal tubulopathy such as Bartter syndrome or Fanconi syndrome. After 1 month of receiving Polycitra, the patient gained 400 g of weight (P3.) and his serum potassium level increased to 3 mmol/L while the blood pH decreased to pH 7.39. However, HCO3 was measured 14.1 mmol/L, urine pH was 6 and glucosuria, proteinuria, and ketonuria persisted. After 6 months of treatment, serum potassium was still below the normal range with 3.2 mmol/L. The patient showed mild hypophosphatemia (3.5 mg/dl) (normal range 4–7 mg/dl).

**Figure 1:**
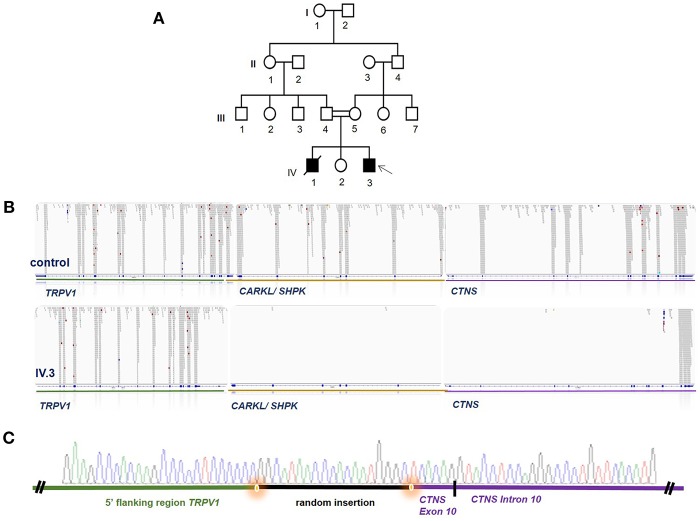
WES identifies a homozygous deletion encompassing large parts of CTNS. **(A)** Pedigree of the Iranian family, DNA of individual IV3 was analyzed by WES (arrow) while no DNA of other family members was available. Females are marked with circle, males with square, solid filling marks affected individuals, crossed symbols marks a deceased individual, double lines between individuals mark a consang. marriage and single lines mark non-consang.-marriages. **(B)** WES revealed a large genomic deletion for individual IV.3 encompassing parts of TRPV1, all of CARKL/SHPK and large parts of CTNS as visualized in the BAM file generated from WES data. Read alignment of a control person is shown above for comparison. **(C)** Breakpoint confirmation by Sanger sequencing confirms presence of the homozygous deletion and reveals a random insertion shown in black.

An older sibling who passed away previously at the age of 10 years had a medical history of renal transplant rejection, however the underlying cause of ESRD had not been clearly defined. An 8 years old sister did not show any kidney or eye problems (Figure 1). We therefore proceeded to perform WES to establish a molecular diagnosis to optimize the therapeutic approach in knowledge of the underlying condition as well as in light of being able to offer genetic counseling for the family.

WES data filtering was performed as described in the Methods section of this manuscript. No promising homozygous variant in known tubulopathy genes were identified amongst the SNVs or INDELs remaining after filtering. We therefore proceeded to inspect the BAM file for homozygous deletions in known tubulopathy genes and, somewhat surprisingly, detected a large homozygous deletion encompassing the *CTNS* gene (Figure 1). Sanger sequencing confirmed this deletion which spans from exon 10 of the *CTNS* gene to intron 2/3 of *TRPV1*, encompassing *CARKL/SHP* but sparing any coding sequence of *TRPV1* gene (Figure 1). This deletion has been previously reported as European founder allele but has not been detected in Middle-Eastern cystinosis patients before. Unfortunately, no DNA of the parents of the index case or the deceased sibling was available for genetic testing.

## Discussion

We describe a case clinically presenting with proximal renal tubular dysfunction as suggested by proteinuria and glucosuria as well as polydipsia, polyuria, vomiting, failure to thrive, and mild hypokalemia. However, blood gas analysis revealed very mild alkalosis, likely with a respiratory component possibly due to screaming of the child during blood draw as indicated by low CO2 in combination with low HCO3–. This caused confusion amongst the admitting local clinicians with regards to the precise underlying condition of the tubular disore as (metabolic) alkalosis would rather be compatible with Bartter Syndrome while metabolic acidosis would be expected in nephropathic cystinosis. Mild hypophosphatemia was noted at a repeat hospital visit in Iran but the patient never developed a clinical picture of rickets. In combination with limited clinical information of his older sibling who passed away after he developed ESRD, we decided to perform WES as first line diagnostic test instead of single gene analyses of known Bartter and renal fanconi syndrome genes. This resulted in the identification of the previously described homozygous 57-kb deletion comprising nearly the entire *CTNS* gene and led to the clinical diagnosis of infantile nephropathic cystinosis in the family. This allele is very frequent in Northern European/Northern American populations of European ancestry due to a German founder mutation originating around 500 AD (15). In other populations this allele is less often identified and previously published reports suggested it doesn't contribute to Cystinosis in the Middle East. However, largely unavailable genetic testing and incomplete clinical data in developing countries creates a significant gap in the rate of cystinosis diagnosis between developed and developing countries (16).

**Figure 2:**
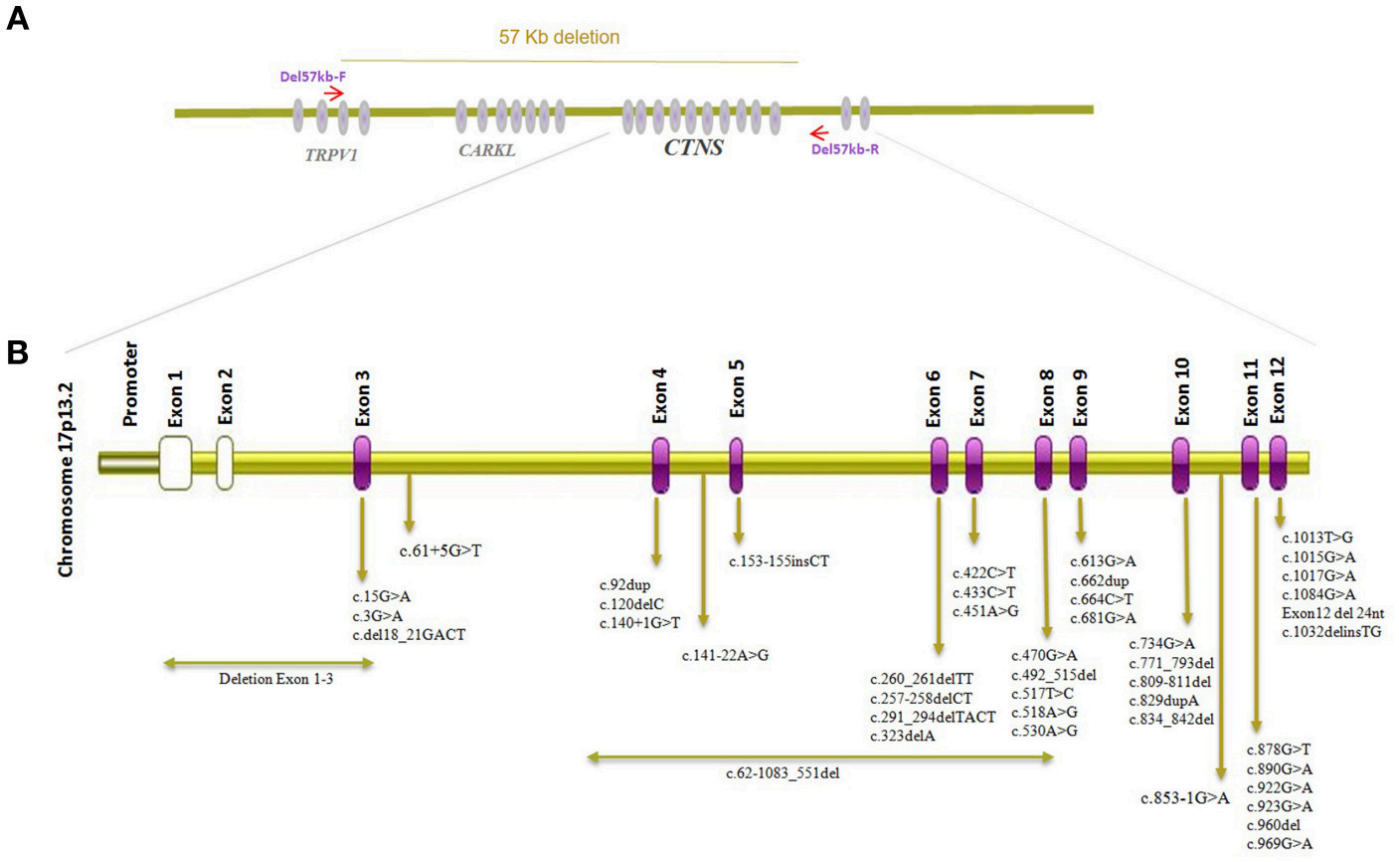
*CTNS* gene structure and visualization of alleles detected in individuals of Middle-Eastern ethnical origin. **(A)** Visualization of the genomic location of *CTNS* and genomic breakpoints of the 57 kb deletion. **(B)** Localizations of CTNS alleles described in cystinosis patients from the Middle East.

*CTNS* mutation surveys of Cystinosis in the Middle East population including Iran, Turkey, Saudi Arabia, Egypt, and Jordan have been summarized in Table 1 with mutation localization to CTNS on protein level displayed in Figure 2. The most commonly identified CTNS allele in Iranian individuals was the c.681G>A (p.Glu227^*^) variant and together with the c.922G>A (p.Gly308Arg), c.681G>A (p.Glu227^*^) is also the most common variant in the Saudi Arabia population. In Jordan, c.890G>A, p.Trp297^*^ has been identified in more than one family. c.451A>G, p.Arg151Gly, c.681G>A (p.Glu227^*^) and 0.834_842del, p. Val279_Tyr281del are the most common alleles in the Turkish population while in Egypt, c.829dup, p.Thr277Asnfs^*^19 has been reported in 4 different cases. Interestingly, c.681G>A (p.Glu227^*^) is the overall most frequently identified allele detected in all Middle eastern cohorts and most variant represent stop or frameshift alleles (Table 1) (17–22, 26). Studies investigating *CTNS* mutations in Cystinosis patients from the Far East have likewise reported several alleles including compound heterozygous mutation c.329G>C, c.329+2T>C in Japan, c.926G>A, c.850C>T, c.18-21del/c and c.969C>G in Thailand, and compound heterozygous mutations c.329+1del andc.463_464del in China while none of the reported cases carried the 57-kb deletion (27–29) but a smaller deletion encompassing ~20 kb of genomic DNA extending from *CARKL* intron 1 *to CTNS* intron 6, c.771–793del, and a c.1515G>A variant have been reported in Tunisian nephrotic patients (30). The 57-kb deletion has however been previously detected in patients from African American ethnicity (31).

**Table 1:** Summary of previously published CTNS causing variants in patients from the Middle-East.

**Country**	**Patient genotype**	**Number of cases**	**Study**
Jordan	c.890G>A, p.Trp297^*^ homoz.	2 (17)	(17) (1 family in this study was originally from Sudan)
	c.829dupA p.Thr277Asnfs^*^19 het / -	1 (17)	
	c.829dupA, p.Trp297^*^ het / c.890G>A, (p.Thr277Asnfs^*^19)	1 (17)	
	c.829dupA, p.Thr277Asnfs^*^19 homoz.	2 (17)	
Iran	c.681G>A, p.Glu227^*^ homoz.	8 (18), 13 (19)	(18–20)
	c.681G>A, p.Glu227^*^het. / c.1017G>A, p.Gly339^*^het.	2 (18)	
	c.681G>A, p.Glu227^*^het. / c.923G>A, p.Gly308Glu *het*.	1 (18)	
	c.del18_21GACT, p.Thr7Phefs^*^7 *homoz*.	2 (20)	
	c.del18_21GACT, p.Thr7Phefs^*^7 *het*.	1 (18)	
	c.923G>A, p.Gly308Glu *homoz*.	1 (18)	
	c.153-155insCT, p.Ala52Leufs^*^5 *homoz*.	1 (18)	
	c.969C>A, p.Asn323Lys *het*.	4 (20)	
	c.323delA, p.Gln108Argfs^*^10 *homoz*.	3 (20)	
	c.257-258delCT, p.Ser86Phefs^*^38 *homoz*.	2 (20)	
	c.662dup, p.Gln222Alafs^*^6 *homoz*.	1 (20)	
	c.92dup, p.Val32Argfs^*^28 *homoz*.	1 (20)	
	c.120delC, p.Asn41Thrfs^*^10 *homoz*.	2 (20)	
	c.517T>C, p.Tyr173His *homoz*.	1 (19)	
	c.613G>A, p.Asp205Asn *homoz*.	1 (19)	
	c.433C>T, p.Gln145^*^homoz.	1 (19)	
	c.1015G>A, p.Gly339Arg) *homoz*.	1 (19)	
	c.681G>A, p.Glu227^*^het / c.853-12T>C *het*.	1 (19)	
	c.492_515del, p.Leu165_Ala172del *homoz*.	2 (19)	
Egypt	c.922G>A, p.Gly308Arg *homoz*.	1 (21)	(21)
	c.809_811del, p.Ser270del *homoz*.	1 (21)	
	c.15G>A, p.(Trp5^*^) *homoz*.	1 (21)	
	c.681G>A, p.(Glu227^*^homoz.	1 (21)	
	c.260_261delTT, p.(Phe87Serfs^*^37) *het*. / c.560A>G, p.(Lys187Arg) *het*.	1 (21)	
	c.1015G>A, p.(Gly339Arg) *homoz*.	1 (21)	
	c.734G>A, p.(Trp245^*^) *het* / c.1032delinsTG, p.(Phe345Valfs^*^85) *het*.	2 (21)	
	c.1084G>A, p.(Gly362Arg) *het*.	1 (21)	
	c.61+5G>T *homoz*.	1 (21)	
	c.829dup, p.(Thr277Asnfs^*^19) *homoz*.	4 (21)	
Turkey	c.451A>G, p.Arg151Gly *het*./ c.1015G>A, p.(Gly339Arg *het*.	2 (22), (23)	(14, 22–25)
	c.del18_21GACT, p.Thr7Phefs^*^7 *homoz*.	3 (22), (23)	
	c.140+1G>T *homoz*.	1 (22)	
	c.518A>G, p.Val171Ala *homoz*.	1 (22)	
	c.681G>A, p.Glu227^*^ het / c.1015G>A, p.Gly339Arg *het*.	1 (22)	
	c.681G>A, p.Glu227^*^homoz.	10 (22), (23), (24)	
	c.del18_21GACT, p.(Thr7Phefs^*^7) het/ c.470G>A, p.(Gly157Asp) *het*.	1 (22)	
	c.62-1083_551del *homoz*.	1 (22)	
	c.451A>G, p.Arg151Gly *homoz*.	10 (22, 23)	
	c.141-22A>G *het*/ c.681G>A, p.Glu227^*^het.	2 (22)	
	c.834_842del, p. Val279_Tyr281del *homoz*.	6 (23)	
	c.325_329del, p.(Thr109Profs^*^14) *homoz*	1 (24)	
	c.960del, p.Tyr321Thrfs^*^8 *homoz*.	1 (14)	
	c.853-1G>A *homoz*.	1 (14)	
	c.664C>T, p.Gln222^*^homoz.	1 (14)	
	c.291_294delTACT, p.Thr98Phefs^*^19 *homoz*.	1 (14)	
	c.3G>C, p.Met1Ile *het*.	1 (14)	
	c.878G>T, p.Ser293Ile *het*.	1 (14)	
	c.853-1G>A *homoz*.	1 (25)	
Saudi Arabia	c.422C>T, p.Ser141Phe *homoz*.	5 (26)	(26)
	Deletion exon 1-3 *homoz*.	1 (26)	
	c.530A>G, p.Asn177Ser het / c.1013T>G, p.Leu338Arg *het*.	2 (26)	
	c.681G>A, p.Glu227^*^homoz.	3 (26)	
	c.922G>A, p.Gly308Arg *homoz*.	1 (26)	
	c.1013T>G, p.Leu338Arg *homoz*.	6 (26)	
	Exon12 del 24nt *homoz*.	1 (26)	

Currently, in Iran as well as many other developing countries, cystinosis is most often diagnosed by slit lamp examination, however corneal crystals may not develop until 1–1.5 years of age. There is therefore a possibility that renal disease may be present before corneal abnormalities can be noted and a critical treatment window is left unused. Renal cystinosis symptoms can closely resemble findings in other tubulopathic diseases such as Dent‘s disease, Lowe’s syndrome, Bartter syndrome and nephrogenic diabetes insipidus and without corneal findings, the correct diagnosis of cystinosis may not be achieved (32). Therefore, additional easy to access diagnostic tests are required, including genetic evaluation. Our study highlights the importance to specifically investigate this allele in non-European populations, including subjects from Iran. Exome data offers great possibilities to detect homozygous alleles by visual BAM file inspection, however heterozygous alleles will be missed by this method and require CNVs analysis. Nevertheless, slit lamp examination of the eyes should be used as initial diagnostic test in patients with clinical suspicion of cystinosis.

As the 57-kb deletion encompasses *CARKL/SHPK* encoding sedoheptulokinas enzyme, a recent study has also suggested measuring activity of the encoded sedoheptulokinas enzyme if cystinosis is suspected. This test however is not applicable for patients with other CTNS mutations (33) and given the rarity of the phenotype, may only be available in large centers. Genetic workup therefore remains currently the most reliable and feasible diagnostic method with whole exome sequencing costs below 500 Euros per sample and available through a number of commercial companies and Sanger sequencing being even cheaper.

In summary, while previous studies suggested that the 57.kb deletion does not play a role in the Middle East, in fact this allele also needs to be taken into consideration when screening *CTNS*, particularly in Iranian patient with Persian ethnicity. As corneal findings may occur after the onset of renal symptoms which in turn share common characteristics with other tubulopathies, we suggest to perform WES or NGS gene panel sequencing as a cost- and time-efficient genetic workup to secure the diagnosis and enable genetic counseling and prenatal diagnosis.

## Ethics Statement

Genetic analysis was conducted under the Innovative Diagnostics Program Human Genetics Department, Radboudumc Nijmegen, The Netherlands.

## Author Contributions

MN and MS wrote the manuscript. MN performed WES analysis and Sanger sequencing. ZB and DA were involved in WES logistics and sample preparation. IS was involved in assessment of clinical data. DMKT, AA, FB, SS-B, and EGK were involved in patient recruitment and clinical care. MS supervised the work and provided funding. MS, MN, and DMKT designed the study.

### Conflict of Interest Statement

The authors declare that the research was conducted in the absence of any commercial or financial relationships that could be construed as a potential conflict of interest.
